# Left Ventricular Longitudinal Strain Detects Ischemic Dysfunction at Rest, Reflecting Significant Coronary Artery Disease

**DOI:** 10.3390/diagnostics15091102

**Published:** 2025-04-26

**Authors:** George Koulaouzidis, Panagiota Kleitsioti, Maria Kalaitzoglou, Christos Tzimos, Dafni Charisopoulou, Panagiotis Theodorou, Ioannis Bostanitis, Adam Tsaousidis, Vasileios Tzalamouras, Pinelopi Giannakopoulou, Aggeliki D. Mavrogianni, Michael Y. Henein, John Zarifis

**Affiliations:** 1 Department of Biochemical Sciences, Pomeranian Medical University, 70-204 Szczecin, Poland; 2 Cardiology Department, General Hospital G. Papanikolaou, 57010 Thessaloniki, Greece; pennykleitsioti@yahoo.com (P.K.); mariakalaitzo25@gmail.com (M.K.); theod80@hotmail.com (P.T.); bostangiannis@yahoo.gr (I.B.); adamtsaou@hotmail.com (A.T.); vtzalamouras@yahoo.gr (V.T.); giannakpop@hotmail.com (P.G.); amavrogianni@yahoo.com (A.D.M.); zarifis.john@gmail.com (J.Z.); 3 Hellenic Statistical Authority, 54646 Thessaloniki, Greece; ctzimos@gmail.com; 4 Paediatric Cardiology Department, Great Ormond Street Hospital, London WC1N 3JH, UK; dafnithess@yahoo.co.uk; 5 National Heart and Lung Institute, Imperial College, London SW3 6LY, UK; henein@gmail.com

**Keywords:** myocardial strain, speckle-tracking echocardiography, global longitudinal strain, stable coronary artery disease, coronary angiogram

## Abstract

**Background/Objectives:** The role of speckle-tracking echocardiography in the diagnosis of stable coronary artery disease (CAD) remains controversial. The aim of this study was to assess the diagnostic accuracy of global longitudinal strain (GLS) in predicting significant CAD. **Methods:** In this prospective study, 103 symptomatic patients referred for invasive coronary angiography were enrolled. All patients underwent resting echocardiography with GLS assessment prior to angiography. Exclusion criteria included acute coronary syndrome, known history of CAD, and the presence of left ventricular wall motion abnormalities. Significant CAD was defined as ≥50% stenosis in at least one major epicardial coronary artery. **Results:** The mean patient age was 63.8 ± 9.3 years, with 78.6% being male. Hypertension was present in 63.1% of patients, dyslipidemia in 77.7%, diabetes mellitus in 22.3%, smoking history in 71.9%, and a family history of premature CAD in 24.3%. Significant CAD was identified in 45.6% (*n* = 47), while the remaining 54.3% (*n* = 56) had non-significant or no coronary artery disease. Patients with significant CAD exhibited significantly lower GLS values compared to those without (−15.73 ± 2.64% vs. −17.6 ± 1.85%, *p* = 0.001). A GLS threshold of >−16.3 predicted significant CAD with 66% sensitivity and 73.2% specificity (AUC = 0.692, *p* = 0.001). GLS demonstrated diagnostic accuracy in identifying disease in individual coronary territories, with AUCs of 0.754 for the left anterior descending artery (LAD), 0.714 for the left circumflex artery (LCx), and 0.723 for the right coronary artery (RCA). Diagnostic performance improved when GLS was combined across all three territories (AUC = 0.796). **Conclusions:** Resting myocardial GLS is accurate in detecting ischemic myocardial dysfunction and can accurately predict significant stenosis of the respective coronary branch subtending the segments.

## 1. Introduction

Coronary artery disease (CAD), remains the leading cause of death worldwide, placing a substantial burden on personal health, the economy, and the society as a whole [1,2]. With its multifactorial etiology, CAD is a complex condition influenced by risk factors such as hypertension, diabetes, hyperlipidemia, and lifestyle choices, including smoking and physical inactivity. Thus, accurate and early detection of CAD is crucial for preventing adverse outcomes such as myocardial infarction, heart failure, and sudden cardiac death [3,4]. Early diagnosis enables timely intervention, which can significantly improve patient outcomes and reduce healthcare costs.

Echocardiography is the primary cardiac imaging modality for individuals with suspected heart pathology, valvular, myocardial, or pericardial, and is one of the first-line investigations for those presenting with chest pain [5]. The suspicion of CAD may be raised when left ventricular systolic (LV) function is reduced and/or there are regional wall motion abnormalities [6]. However, most patients presenting with chest pain frequently have preserved LV ejection fraction (EF) [7,8]. This underscores the need for more sensitive imaging techniques to detect early myocardial dysfunction. Recently developed echocardiographic techniques, such as myocardial Doppler imaging and speckle-tracking echocardiography (STE), allow further evaluation of different components of myocardial contraction (radial, longitudinal, and circumferential), thus enabling the assessment of both global and regional systolic function [9,10].

Global longitudinal LV strain (GLS), which measures active LV cavity shortening in the longitudinal direction, can detect subtle changes in myocardial function even in the absence of significant conventional wall motion abnormalities [10,11,12]. Despite being sensitive in detecting myocardial dysfunction, inconsistencies exist regarding GLS accuracy in predicting CAD. Much of the existing data are based on stress GLS as an adjunct to conventional diagnostic techniques, e.g., stress echocardiography and exercise treadmill test, without fully evaluating its potential as an independent diagnostic tool [13,14]. Furthermore, there is considerable variation in reporting, and limited consensus on the optimal GLS cut-off values for predicting significant CAD or distinguishing between single-vessel and multi-vessel disease [15,16,17,18].

The aim of this study is to evaluate the accuracy of LV best GLS cut-off value, at rest, in predicting the presence of significant CAD (≥50% stenosis of epicardial coronary arteries) in symptomatic patients without prior history of CAD.

## 2. Materials and Methods

This is a prospective observational study conducted at a single tertiary heart center. Consecutive patients with angina pectoris who were referred for coronary angiography to assess CAD were considered for inclusion in the study. The study was approved by the institution ethics committee (reference number 156) and was conducted in accordance with the Declaration of Helsinki.

Inclusion criteria: Patients were considered eligible for inclusion in the study based on predefined criteria. The inclusion criteria required that patients be aged 18 years or older, regardless of sex, with a clinical presentation and history suggestive of stable angina. These patients were also required to have preserved LV function defined as an EF > 50% and also without regional wall motion abnormalities on two-dimensional (2D) echocardiography.

Exclusion criteria: Patients were excluded from the study if they exhibited any of the following characteristics: LVEF below 50% on 2D echocardiography, a known history of CAD, previous percutaneous coronary intervention (PCI), prior coronary artery bypass graft (CABG) surgery, or heart failure. Additionally, patients were excluded if they had more than mild valvular heart disease or presented with acute coronary syndrome (ACS), as confirmed by significantly elevated cardiac enzyme levels (serum troponin) or the presence of pathological Q waves on resting electrocardiography (ECG).

All patients underwent a comprehensive medical history review, including demographic data collection such as age, gender, and body mass index (BMI). Common cardiovascular risk factors (smoking status, diabetes, hypertension, dyslipidemia, family history of premature CAD) were also assessed. Additionally, the presence of any associated comorbidities was recorded. A detailed cardiac examination and resting ECG were performed for all patients. The data collected were documented in a pre-designed data collection form, ensuring that all patient-specific information remained strictly confidential and was used solely for research purposes.

A standard 2D transthoracic echocardiogram (TTE) was performed with patients positioned in the left lateral decubitus posture. Echocardiographic imaging was conducted using a commercially available system (VIVID 7, General Electric Vingmed Ultrasound, Milwaukee, WI, USA), equipped with either a 3.5 MHz or an M5S transducer. Two experienced echocardiologists carried out the echocardiographic examinations. The echocardiographic protocol and measurements adhered to the recommendations for chamber quantification as established by the American Society of Echocardiography and the European Association of Cardiovascular Imaging. Additionally, the protocol followed expert consensus guidelines set forth by the European Association of Cardiovascular Imaging. All echocardiographic images were digitally stored and subsequently analyzed offline using EchoPac version 12 (GE Healthcare, Chicago, IL, USA) by a single investigator who was blinded to all baseline clinical and angiographic data. The left ventricular end-diastolic and end-systolic volumes were calculated using the Simpson’s biplane method, based on apical two-chamber and four-chamber views. The LVEF was derived from these measurements.

Two-dimensional speckle-tracking analysis was performed in the apical two-chamber, three-chamber, and four-chamber views to calculate GLS. The endocardial border was manually traced at end-systole, and the width of the region of interest (ROI) was manually adjusted to include the entire myocardial wall thickness. Three cardiac cycles were analyzed, and the measurements were averaged. The integrity of speckle tracking was automatically detected and visually ascertained. The 2D speckle-tracking GLS analyses were performed on LV greyscale images (VersionBT13, GEMedical Systems). Peak GLS was measured from the 17 segment measurements (six segments from each of the apical four-, two-, and three-chamber views). Any poorly read segments were automatically excluded by the software.

On the same day as the echocardiographic examination, each patient underwent coronary angiography utilizing the Judkins technique. Angiographic images were obtained in multiple projections for each coronary artery. The extent of coronary artery stenosis was visually estimated by an experienced operator who was blinded to the echocardiographic findings. A luminal narrowing of ≥50% in any of the major coronary arteries, including the left anterior descending artery (LAD), left circumflex artery (LCX), and right coronary artery (RCA), was considered significant CAD. Fractional Flow Reserve (FFR) was performed in a subset of eight patients to assess the physiological significance of coronary stenosis. This measurement was carried out using a pressure wire to determine the ratio of distal coronary pressure to proximal aortic pressure during induced hyperemia.

Statistical analysis: All statistical analyses were conducted using the IBM SPSS Statistics software (version 25), with the level of statistical significance set at *p* < 0.05. To examine differences in the means of independent variables, a t-test was applied, after verifying the necessary assumptions of normality in distributions and homogeneity of variances between groups. For the analysis of 2 × 2 contingency tables, Fisher’s exact probability test was used for a two-tailed test. Also, the size effect was assessed using Cohen’s d for quantitative variables and Cohen’s w for qualitative variables. The sensitivity and specificity calculations were performed using the Receiver Operating Characteristic (ROC) curve, and Logistic Binary Regression was applied to compute the Relative Risk for each variable (e.g., coronary arteries) and the corresponding GLS percentages.

## 3. Results

A total of 103 subjects were enrolled in the study based on the established inclusion and exclusion criteria. The mean age of the study population was 63.7 ± 9 years, 81 (78.6%) of whom were males. Regarding risk factors for coronary disease, 23 subjects (22.3%) were diabetics, 26 (25.2%) were obese, 47 (45.6%) smoked, 65 (63.1%) had hypertension, and 80 (77.7%) had dyslipidemia. Additionally, 23 subjects (22.3%) had a family history of premature CAD, 6 (5.8%) had chronic kidney disease, 12 (11.7%) had peripheral arterial disease, and 9 (8.7%) had atrial fibrillation (AF).

Based on the coronary angiogram results, 56 (54.4%) subjects had <50% CA stenosis; hence, they were stratified as having non-significant CAD (nsCAD). The remaining 47 (45.6%) subjects had more than one CA with ≥50% stenosis; thus, they were classified as having a significant CAD group. Of these patients, 27 (26.2%) had single-vessel disease, 13 (12.6%) had two-vessel disease, and 7 (6.8%) had three-vessel disease.

The demographic data of the included subjects showed significantly more females in the nsCAD group. Individuals’ age and other risk factors were comparable between the two groups with and without coronary stenosis (Table 1).

**Figure 1:**
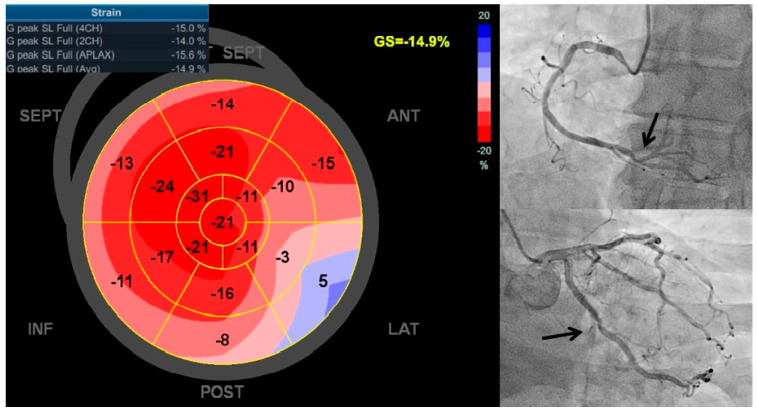
A 64-year-old male presented with exertional chest pain and was referred for evaluation of suspected coronary artery disease. Resting echocardiography showed preserved left ventricular ejection fraction (LVEF), but a markedly reduced GLS of 14.9% (**image on the left**). Coronary angiography revealed a 70% stenosis in the right coronary artery (**image on the right top**) and complete (100%) occlusion of the left circumflex artery (**image on the right bottom**).

**Figure 2:**
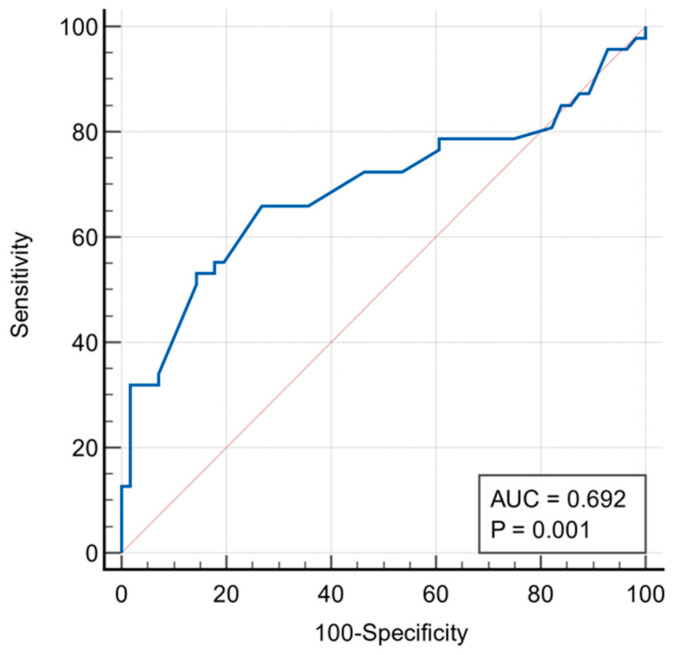
ROC curve of GLS value to predict the presence of significant coronary artery disease (CAD).

Although the LVEF did not differ significantly between the two patients groups, mean LV GLS was significantly lower in patients with significant CAD compared to those with nsCAD (−15.9 ± 2.7 vs. −17.5 ± 1.84%, *p* = 0.0008), as seen in Figure 1. This difference was significant irrespective of the number of diseased coronary arteries; single-vessel disease (−16.2 ± 2.4%, *p* = 0.01), two-vessel disease (−15.5 ± 2.5%, *p* = 0.02), and three-vessel disease (−13.9 ± 2.5%, *p* = 0.008). Notably, the number of diseased vessels had a progressively greater impact on the reduction in LV GLS (*p* < 0.0001). A GLS cut-off value of >−16.3 successfully predicted the presence of significant CAD with a sensitivity of 66% and a specificity of 73.2% (AUC: 0.692, *p* = 0.001), as shown in Figure 2.

The diagnostic accuracy of GLS in detecting coronary artery stenosis is shown in Table 2. Varying degrees of diagnostic accuracy were observed among individual coronary arteries. GLS showed an accuracy of 0.754 (AUC) for diagnosing LAD stenosis, 0.714 for LCx, and 0.723 for RCA. When GLS measurements were combined for LAD, LCx, and RCA, the diagnostic accuracy became significantly higher (AUC = 0.796). The highest diagnostic performance was observed when GLS was combined with dyslipidemia, yielding an AUC of 0.868 (95% CI: 0.800–0.937).

The multiple regression analysis identified dyslipidemia as the only significant cardiovascular risk factor associated with reduced GLS, with a *p*-value of 0.044, thus indicating a direct relationship (Table 3). In contrast, diabetes mellitus, current smoking, ex-smoking, and family history did not demonstrate any significant impact, with *p*-values of 0.071, 0.15, 0.119, and 0.65, respectively.

The relationship between LV GLS and the relative risk (RR) of having significant coronary artery stenosis in the main epicardial arteries is seen in Table 4. The data clearly show that as GLS values become more negative, the risk of severe coronary stenosis increases significantly. At GLS = −11.0, the probability of significant LAD stenosis is 89.2%, while the likelihood of having significant stenosis in any of the three main arteries (LAD, LCx, or RCA) rises to 97.2%. Furthermore, when dyslipidemia is also present, the risk escalates to 99.8%, thus highlighting the additional burden of dyslipidemia on CAD. Conversely, as GLS values become less negative, the relative risk decreases progressively. At GLS = −21.5, the risk of having LAD stenosis drops to 9.4%, and the overall risk when considering dyslipidemia is only 11.4%; its role is a critical marker for identifying patients at high risk of significant stenosis (Table 4).

## 4. Discussion

Findings: This study evaluated the accuracy of global longitudinal strain in predicting significant coronary artery disease. The results indicate that GLS is a reliable marker for detecting myocardial dysfunction in patients with significant CAD stenosis. Mean GLS was lower in patients with significant CAD compared to those with non-significant CAD. Furthermore, GLS values showed a progressive decline with the increased severity of CAD, with GLS of −16.2 ± 2.4% for single-vessel, −15.5 ± 2.5% for two-vessel, and −13.9 ± 2.5% for three-vessel disease. A GLS cut-off value of >−16.3 predicted significant CAD with a sensitivity of 66% and specificity of 73.2% (AUC: 0.692, *p* = 0.001). This diagnostic accuracy varied in individual coronary arteries, with AUC of 0.754 for LAD, 0.714 for LCx, and 0.723 for RCA disease. But the diagnostic accuracy of GLS increased significantly (AUC = 0.796) in patients with three-vessel disease. A GLS of −11.0 carries a likelihood for significant stenosis in any of the three main arteries of 97.2%. Multiple regression analysis identified dyslipidemia as the only CV risk factor significantly associated with GLS. In contrast, diabetes mellitus, smoking, and family history of CAD did not exhibit any significant associations.

Interpretation of the Results: The above findings indicate that LV GLS is a much more sensitive marker for significant coronary disease compared with the conventionally used LV EF, which was normal in all our patients. The latter is an estimate of global LV cavity systolic fall in volume with respect to its diastolic volume. However, LV GLS assesses specifically the extent of systolic myocardial shortening with respect to its diastolic length, thus is considered a reflector of intrinsic myocardial function. This function is particularly sensitive to reduced myocardial perfusion resulting from significant coronary stenosis [19]. This relationship has been shown to become tighter during stress and increase in heart rate, as myocardial oxygen demand increases [20]. Of course, additional distal microcirculation disease causing reduced myocardial vascularization adds to the compromised blood supply and its impact on segmental systolic function. Furthermore, LV GLS assesses mainly longitudinal myocardial systolic function, which is conducted by the longitudinal myocardial fibers forming the subendocardial layer of the myocardium [21]. This layer is well known to be the most sensitive to ischemia and compromised blood supply due to the above-mentioned mechanisms [22].

Our findings also highlight the importance of LV GLS in reflecting disturbed ‘ischemic’ segmental function at rest in the absence of symptoms or ECG abnormalities. Although this may not support the conventional pathological definition of ischemia, it cannot be explained otherwise. In fact, the same pattern of reduced LV GLS in patients with significant CAD has been shown to develop during stress-induced ischemia [23] and to recover afterwards, thus supporting the fact that our findings are respective of those, but at rest. The significant increase in LV GLS accuracy in predicting CAD stenosis in patients with triple-vessel CAD further supports the above claim about GLS accuracy in demonstrating myocardial ischemic dysfunction at rest. It should be mentioned that the GLS disturbances we hereby report are supported by previously reported LV long axis function studied by M-mode echocardiography, which showed a significant fall in its amplitude of motion, as well as systolic and diastolic velocities during induction of acute ischemia during coronary artery balloon inflation [24]. In addition, all those abnormalities completely recovered within 48 h of successful revascularization, thus further confirming their ischemic explanation [25]. Finally, in our analysis of the impact of conventional atherosclerosis risk factors on the accuracy of GLS in predicting significant CAD, dyslipidemia proved to be the sole risk factor of significant impact. This finding aligns with current guidelines recommendations for optimum management of CAD [26].

Clinical Implications: The ability of GLS to detect significant coronary artery stenosis has important implications for clinical practice. Unlike conventional echocardiographic parameters that rely on visual assessment of wall motion abnormalities, GLS offers a quantitative and reproducible approach to detecting myocardial impairment. Thus, it stands as a valuable echocardiographic parameter for screening newcomers with suspected CAD as a cause for angina-like symptoms.

With coronary angiography being an invasive procedure, the role of non-invasive techniques like echocardiography in identifying high-risk patients is invaluable. Early identification of those at risk for significant CAD can lead to timely interventions, potentially reducing the incidence of adverse outcomes such as myocardial infarction and heart failure. Its accuracy is justified by its reflection of the longitudinal subendocardial myocardial fibers, which are known to be vulnerable to ischemia. In addition, GLS accuracy increases in patients with significant multivessel CAD and dyslipidemia.

The relative risk analysis further demonstrated that as GLS values become more negative, the likelihood of significant coronary stenosis increases markedly. At a GLS value of −11.0, the probability of LAD stenosis ≥ 50% reached 89.2%, whereas the risk of significant stenosis in the three main coronary arteries was 97.2%. When dyslipidemia was present, this risk escalated to 99.8%. Conversely, at GLS values of −21.5, the risk of LAD stenosis and overall CAD involvement was substantially lower, at 9.4% and 11.4%, respectively. This makes it particularly useful in borderline cases, where traditional echocardiographic findings may be inconclusive.

Although our primary analysis focused on GLS, segmental longitudinal strain data were also recorded. Preliminary observations indicated that segments subtended by significantly stenosed coronary arteries often exhibited reduced strain values, in line with ischemic dysfunction. While this segmental analysis was beyond the scope of the current study, it supports the pathophysiological link between localized ischemia and regional myocardial deformation. Future studies from our group aim to explore this aspect in more detail.

Another key advantage of GLS is its cost-effectiveness. While advanced imaging modalities such as cardiac magnetic resonance imaging (MRI) and positron emission tomography (PET) offer high diagnostic accuracy, they are often limited by high costs and restricted availability. GLS, on the other hand, can be easily integrated into routine echocardiography using standard ultrasound machines with speckle-tracking software, making it a practical and accessible tool for widespread clinical use.

Study Limitations: Despite the above promising results, this study has some limitations. Firstly, it was conducted at a single tertiary care center, which may limit the generalizability of the findings to other populations. Secondly, the sample size was relatively small, which may have influenced the statistical power of some comparisons. Another limitation of our study is the absence of tissue Doppler-derived strain rate data, particularly peak systolic strain rate, which has been shown to be a superior indicator of myocardial contractility due to its higher temporal resolution and angle dependency characteristics [27]. Finally, the findings are applicable only to patients without prior known CAD or events.

## 5. Conclusions

In conclusion, GLS appears to be a promising tool for identifying significant CAD, with potential clinical implications in improving early detection and risk stratification. A GLS of −11.0 carries a likelihood of significant stenosis in any of the three main arteries of 97.2%. Future studies with larger cohorts and standardized GLS cut-off values are necessary to further validate these findings and enhance the clinical applicability of GLS in routine CAD assessment.

## Figures and Tables

**Table 1:** Comparison of the basic characteristics of patients with and without significant coronary artery disease (CAD).

Variable	Non-Significant CAD *n* = 56	Significant CAD *n* = 47	*p*-Value
Age (y)	63.9 ± 9.5	63.5 ± 9.2	0.840
Gender (F) (*n* = 22)	17 (30.36%)	5 (10.64%)	0.017
DM (*n* = 23)	13 (23.21%)	10 (21.28%)	0.814
HTN (*n* = 65)	37 (66.07%)	28 (59.57%)	0.543
Dyslipidemia (*n* = 70)	38 (67.86%)	32 (68.09%)	0.980
Smoking (*n* = 47)	24 (42.86%)	23 (48.94%)	0.537
Family history of CAD (*n* = 23)	11 (19.64%)	12 (25.53%)	0.488
Obesity	14 (25%)	12 (25.5%)	0.480
Kidney disease (*n* = 6)	3 (5.36%)	3 (6.38%)	0.825
PAD (*n* = 12)	6 (10.71%)	6 (12.77%)	0.767
	**Echocardiographic**	**Measurements**	
LVEF %	60 ± 5	59 ± 4	0.09
LVEDD, cm	4.6 ± 0.5	4.7 ± 0.6	0.4
LAD, cm	34.5 ± 3.5	35 ± 3	0.09
E/A	1.1 ± 0.3	0.95 ± 0.24	<0.001
E/e′	11.7± 3	10.8 ± 3.2	0.03

**Table 2:** Localization of the affected vessel. LAD: Left anterior descending, LCX: Left circumflex, RCA: Right coronary artery, DLP: Dyslipidemia.

	GLS	Sensitivity	Specificity	AUC (95% C.I.)
LAD	−16.15	0.73	0.78	0.754 (0.653–0.855)
LCx	−15.75	0.71	0.73	0.714 (0.580–0.847)
RCA	−15.45	0.63	0.81	0.723 (0.603–0.843)
LAD and LCx	−16.75	0.73	0.77	0.765 (0.670–0.860)
LAD and RCA	−16.90	0.73	0.82	0.780 (0.689–0.871)
LCx and RCA	−15.75	0.59	0.81	0.707 (0.597–0.816)
LAD and LCx and RCA	−16.90	0.74	0.86	0.796 (0.708–0.884)
LAD and LCx and RCA and DLP	−17.10	0.79	0.96	0.868 (0.800–0.937)

**Table 3:** Multiple regression analysis.

Multiple Regression					
	Unstandardized Coefficients	Standardized Coefficients			
	B	Beta	T	*p*	95.0% C.I. for B
Dyslipidemia	1.144	0.199	2.041	0.044	(0.032–2.256)
Diabetes mellitus	1.029	0.179	1.827	0.071	(−0.089–2.147)
Smoking (current)	0.824	0.171	1.45	0.15	(−0.304–1.952)
Smoking (ex)	1.012	0.186	1.571	0.119	(−0.266–2.29)
Family History	−0.26	−0.045	−0.455	0.65	(−1.394–0.874)

**Table 4:** The relative risk (RR) of significant CAD on individual coronary arteries based on GLS cut-off values.

GLS	LAD	LCx	RCA	LAD and LCx	LAD and RCA	LCx and RCA	LAD and LCx and RCA	LAD and LCx and RCA and DLP
−11.0	89.2%	52.4%	76.2%	92.6%	96.2%	80.3%	97.2%	99.8%
−11.5	87.0%	48.3%	72.6%	91.0%	95.1%	77.5%	96.4%	99.7%
−12.0	84.4%	44.2%	68.6%	89.1%	93.9%	74.4%	95.3%	99.5%
−12.5	81.5%	40.1%	64.3%	86.8%	92.3%	71.1%	94.0%	99.3%
−13.0	78.2%	36.2%	59.8%	84.1%	90.4%	67.4%	92.3%	99.0%
−13.5	74.4%	32.5%	55.2%	81.0%	88.1%	63.6%	90.3%	98.5%
−14.0	70.2%	28.9%	50.4%	77.5%	85.3%	59.6%	87.7%	97.8%
−14.5	65.7%	25.6%	45.6%	73.5%	81.9%	55.4%	84.6%	96.7%
−15.0	60.9%	22.6%	40.9%	69.1%	78.0%	51.2%	80.9%	95.3%
−15.5	55.8%	19.8%	36.4%	64.3%	73.6%	47.0%	76.5%	93.2%
−16.0	50.7%	17.3%	32.1%	59.2%	68.6%	42.8%	71.5%	90.2%
−16.5	45.5%	15.1%	28.1%	53.9%	63.1%	38.7%	65.9%	86.2%
−17.0	40.4%	13.1%	24.4%	48.6%	57.2%	34.7%	59.8%	80.9%
−17.5	35.5%	11.3%	21.0%	43.2%	51.2%	31.0%	53.4%	74.2%
−18.0	30.9%	9.7%	18.0%	38.0%	45.1%	27.4%	46.9%	66.1%
−18.5	26.6%	8.4%	15.3%	33.1%	39.2%	24.2%	40.4%	56.9%
−19.0	22.8%	7.2%	13.0%	28.5%	33.5%	21.2%	34.3%	47.3%
−19.5	19.3%	6.2%	11.0%	24.3%	28.3%	18.5%	28.7%	37.8%
−20.0	16.3%	5.3%	9.3%	20.6%	23.6%	16.1%	23.6%	29.2%
−20.5	13.6%	4.5%	7.8%	17.3%	19.5%	13.9%	19.3%	21.8%
−21.0	11.4%	3.8%	6.5%	14.4%	16.0%	12.0%	15.5%	15.9%
−21.5	9.4%	3.3%	5.4%	11.9%	13.0%	10.3%	12.4%	11.4%
−19.8	17.5%	5.6%	9.9%	22.0%	25.5%	17.0%	25.6%	32.5%
−20.2	15.2%	4.9%	8.6%	19.2%	21.9%	15.2%	21.8%	26.0%
−20.6	13.2%	4.4%	7.5%	16.7%	18.8%	13.5%	18.5%	20.5%
−21.0	11.4%	3.8%	6.5%	14.4%	16.0%	12.0%	15.5%	15.9%

## Data Availability

Due to ethical concerns, supporting data are not publicly available but can be provided upon request with necessary approval.
